# Supplementary material for: Ebola virus exploits host lncRNA LINC01740 to enhance ATF3 and suppress antiviral immune responses
Source: bioRxiv. 2026 Jul 9:2026.07.06.731958. Preprint. [Version 1] doi: 10.64898/2026.07.06.731958 (PMC13370357; doi:10.64898/2026.07.06.731958)
Supplement: Supplement 1 [file NIHPP2026.07.06.731958v1-supplement-1.pdf]

bioRxiv preprint doi: https://doi.org/10.64898/2026.07.06.731958; this version posted July 9, 2026. The copyright holder for this preprint (which was not certified by peer review) is the author/funder, who has granted bioRxiv a license to display the preprint in perpetuity. It is made available under a CC-BY 4.0 International license.

## Supplementary Table S1: Oligonucleotide sequences

| Primers | Oligonucleotide sequences |
|---|---|
| **qPCR primers** | |
| UNARTF | GGGGTCTGTATCTCAAAGCCT |
| UNARTR | TTCCCTTGTGCTCCTTTGCA |
| ATF3RTF | GTCCATCACAAAAGCCGAGG |
| ATF3RTR | GCCGATGAAGGTTGAGCATG |
| IFNβRTF1 | GTCAGAGTGGAAATCCTAAG |
| IFNβRTR1 | ACAGCATCTGCTGGTTGAAG |
| GAPDHRTF | GGTGAAGGTCGGAGTCAACG |
| GAPDHRTR | GTTGAGGTCAATGAAGGGGTC |
| **ChIP-qPCR Primers** | |
| ATF3promoF1 | CATTACGTCAGCCTGGGACT |
| ATF3promoR1 | GGCGAGAGAAGAGAGCTGTG |
| Antisense Oligonucleotide Sequence (ASO) | |
| UNA ASO1 | CATCACCCCAGGAAGTTCCCTTG |
| NC ASO | GGATACTACAACTACAATGGTAC |
| **shRNA sequence** | |
| ATF3 sh | CCTCTTTATCCAACAGATAAA |
| NC sh | GCGCGATGCGCTAATAATTT |
| **guideRNA sequence** | |
| gATF3 | ACAGTGACTGATTCCAGCGCAGA |
| gNT | TCACCAGAAGCGTACCATACTC |
| **Cloning Primers** | |
| XR3MluIF1 | GCCCTACGCGTGAGGGCCTATTTCCCATGATTCCTT |
| XR3BamHIR1 | GAATTGGATCCAAAAAAAAGGTCTTCTCGAAGACCCGTTTCA |
| MSCrev | CAGCGGGGCTGCTAAAGCGCATGC |
| **Sequencing Primer** | |
| PKLV-cpptseqF1 | CAGTGCAGGGGAAAGAATAG |

bioRxiv preprint doi: https://doi.org/10.64898/2026.07.06.731958; this version posted July 9, 2026. The copyright holder for this preprint (w
was not certified by peer review) is the author/funder, who has granted bioRxiv a license to display the preprint in perpetuity. It is made
available under a CC-BY 4.0 International license.

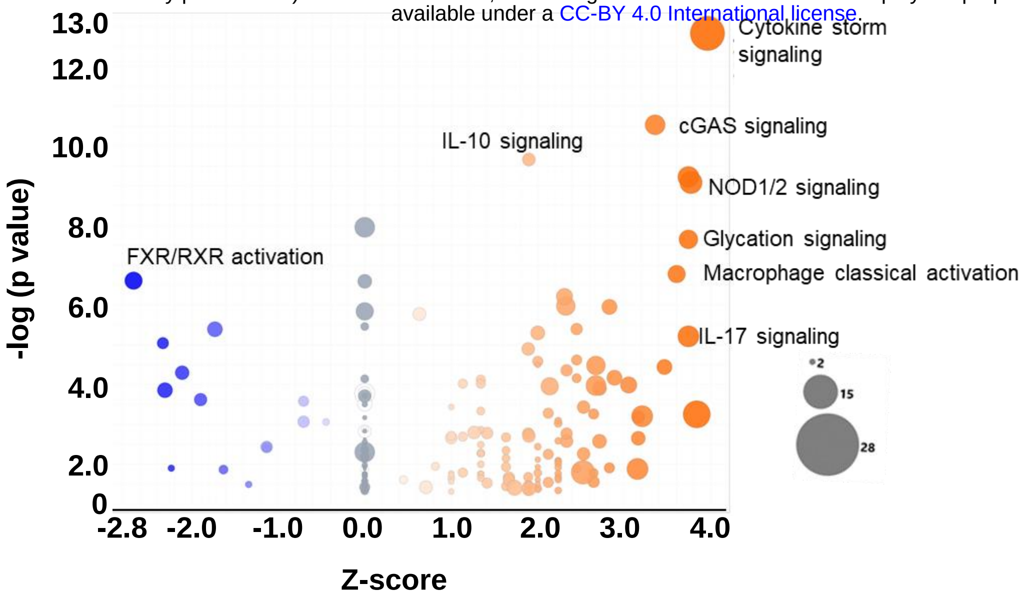


**Supplementary Figure S1. Canonical pathway analysis of EBOV-induced transcriptional responses in macrophages**. Ingenuity Pathway Analysis (IPA) was performed on differentially expressed genes identified by transcriptomic profiling of EBOV-infected versus mock-infected macrophages at 24 h post-infection. The bubble plot displays significantly enriched canonical pathways, with each circle representing a pathway. Bubble size reflects the number of differentially expressed genes mapping to each pathway, and color indicates the direction and relative magnitude of pathway enrichment (blue, negative enrichment; orange, positive enrichment). Significantly regulated pathways are annotated. This analysis highlights robust activation of innate immune and inflammatory signaling programs.

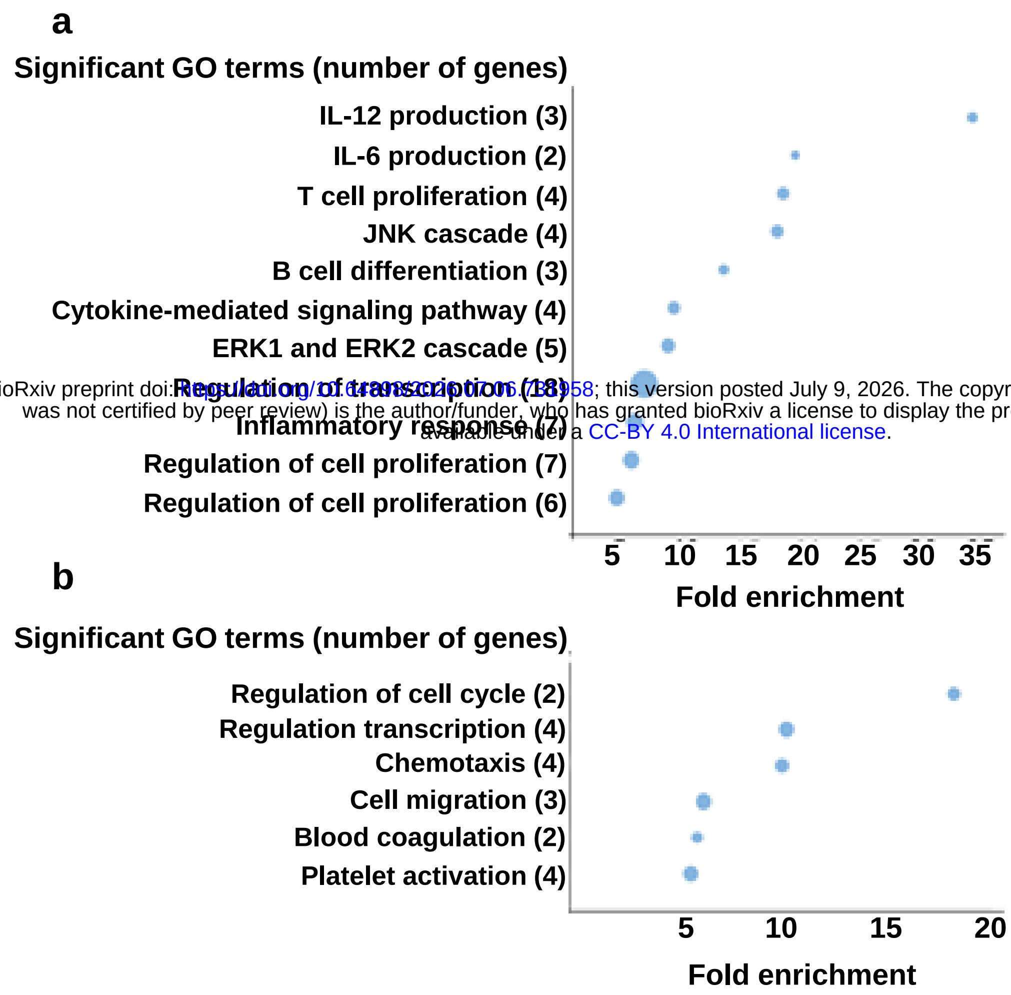


bioRxiv preprint doi: https://doi.org/10.64898/2026.07.06.[illegible]; this version posted July 9, 2026. The copyright holder for this preprint (w
was not certified by peer review) is the author/funder, who has granted bioRxiv a license to display the preprint in perpetuity. It is made
available under a CC-BY 4.0 International license.

**Supplementary Figure S2. Dynamic lncRNA-mRNA Co-regulatory Networks during EBOV Infection.** Protein-coding genes involved in transcriptional machinery and immune responses are co-regulated with differentially expressed lncRNAs at 24 h **(a)** and 48 h **(b)** after EBOV infection in human macrophages. Gene Ontology analysis was performed on protein-coding mRNAs co-regulated with the associated lncRNAs. Significantly enriched pathways are shown, with bubble size indicating the number of genes in each pathway, which are also indicated in parentheses.

bioRxiv preprint doi: https://doi.org/10.64898/2026.07.06.731958; this version posted July 9, 2026. The copyright holder for this preprint (w
was not certified by peer review) is the author/funder, who has granted bioRxiv a license to display the preprint in perpetuity. It is made
available under a CC-BY 4.0 International license.

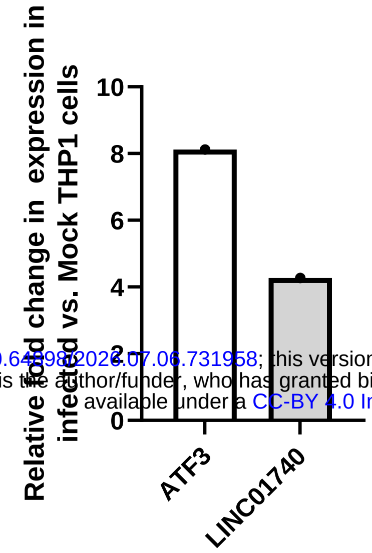


**Supplementary Figure S3. The Makona variant of EBOV induces UNA and ATF3 expression in THP-1 macrophages.** RNA-seq analyses from a previously published study showed significant induction of UNA and ATF3 in PMA-differentiated THP-1 macrophages infected with the Makona variant. Expression of UNA and ATF3 was most significantly increased 72 h post-infection in THP-1 macrophages.

bioRxiv preprint doi: https://doi.org/10.64898/2026.07.06.721958; this version posted July 9, 2026. The copyright holder for this preprint (which was not certified by peer review) is the author/funder, who has granted bioRxiv a license to display the preprint in perpetuity. It is made available under a CC-BY 4.0 International license.

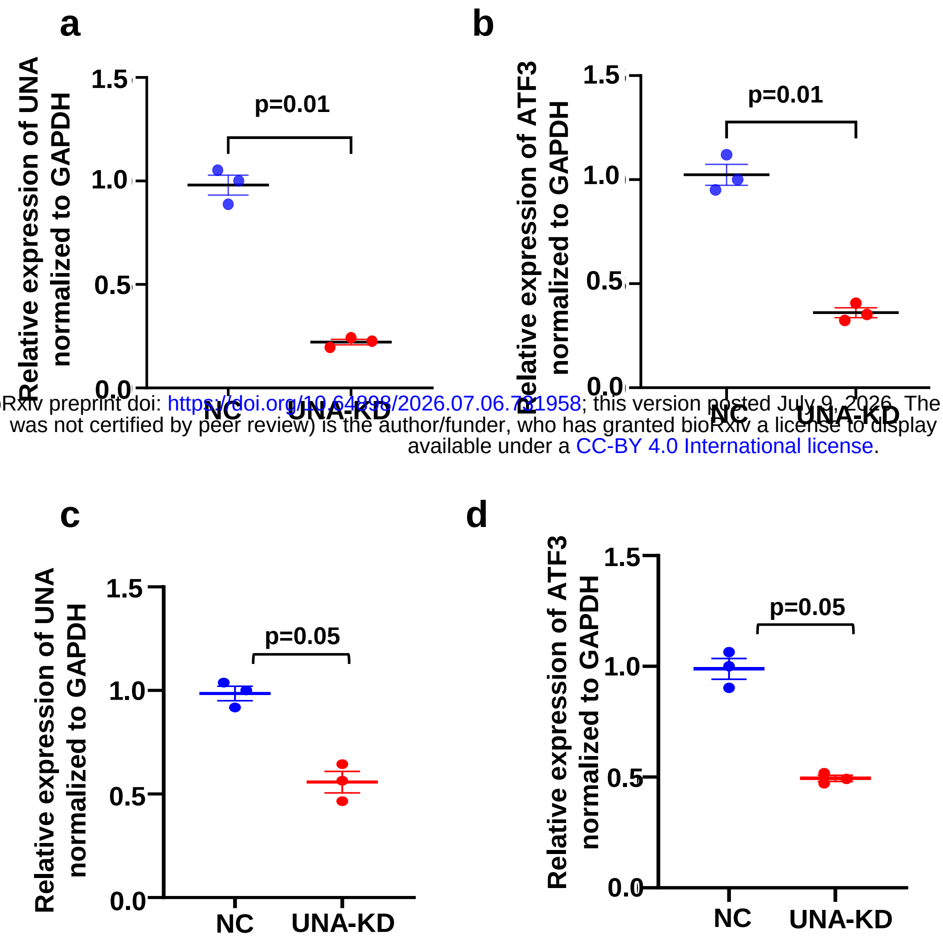


**Supplementary Figure S4. UNA knockdown reduces ATF3 mRNA expression in human cells.** Cells were transfected with an antisense oligonucleotide (ASO) targeting UNA (UNA-KD) or a negative control ASO (NC). **(a)** UNA transcript levels were significantly reduced in LentiX UNA-KD cells compared with NC-transfected cells. **(b)** ATF3 mRNA was also significantly decreased in UNA-KD LentiX cells relative to NC-transfected cells. **(c, d)** Similarly, in HeLa cells, UNA-KD cells showed a significant reduction in UNA and ATF3 transcripts compared with controls (NC). Data are presented as mean ± SEM. Student’s t-test was used for statistical comparison, and a two-tailed p-value is indicated.

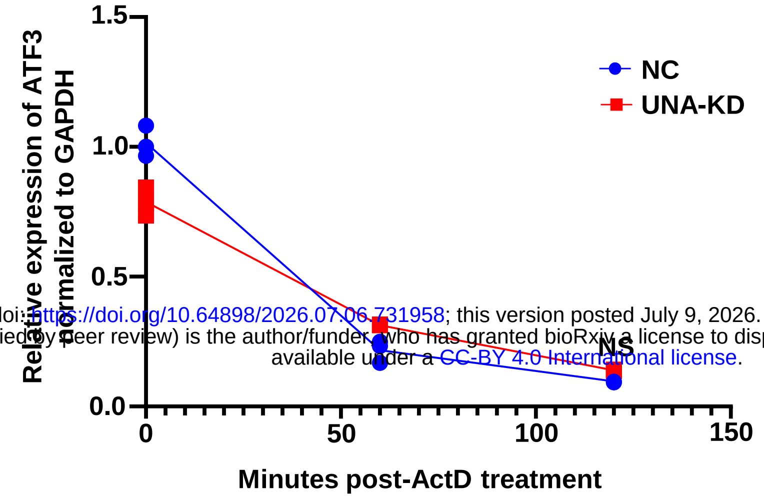


bioRxiv preprint doi: https://doi.org/10.64898/2026.07.06.731958; this version posted July 9, 2026. The copyright holder for this preprint (which was not certified by peer review) is the author/funder, who has granted bioRxiv a license to display the preprint in perpetuity. It is made available under a CC-BY 4.0 International license.

**Supplementary Figure S5 UNA knockdown did not affect ATF3 mRNA stability.** PMA-differentiated THP-1 cells were transfected with an antisense oligonucleotide (ASO) targeting UNA (UNA-KD) or a negative control ASO (NC) for 24 hours. Subsequently, de novo RNA synthesis was blocked with 1 µg/mL actinomycin D. Cells were harvested at the indicated time points, total RNA was extracted, and mRNA expression was measured by qPCR. ATF3 mRNA levels at each time point were compared with those measured before actinomycin D addition. Data are presented as mean ± SEM. Student’s t-test was used for statistical comparison; NS = not significant.

bioRxiv preprint doi: https://doi.org/10.64898/2026.07.06.731958; this version posted July 9, 2026. The copyright holder for this preprint (which was not certified by peer review) is the author/funder, who has granted bioRxiv a license to display the preprint in perpetuity. It is made available under a CC-BY 4.0 International license.

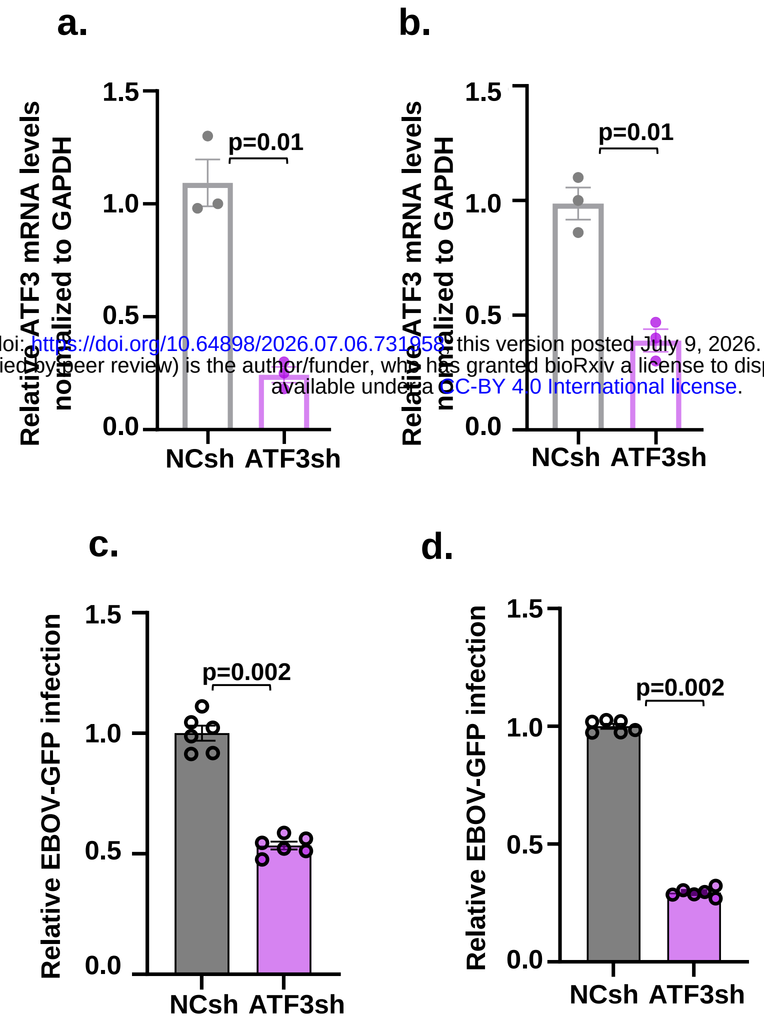


**Supplementary Figure S6. ATF3 knockdown inhibits EBOV infection in HeLa and Huh cells. (a, b)** We used ATF3-targeting shRNAs (ATF3sh) or a negative control (NCsh) to knock down ATF3 expression in HeLa (epithelial) or Huh (hepatocyte) cells. We infected cells with EBOV-GFP at an MOI of 0.2 for 24 h and measured EBOV-GFP+ cells. We observed a significant reduction in EBOV-GFP+ levels in ATF3sh vs. NCsh in both HeLa **(c)** and Huh **(d)** cells. Data are presented as mean ± SEM. Student's t-test was used for statistical comparison.
